# Short-Term Laboratory Outcomes of SGLT2 Inhibitor Use in Type 2 Diabetic Patients: A Retrospective Analysis

**DOI:** 10.3390/jcm14227985

**Published:** 2025-11-11

**Authors:** Hamood AlSudais, Turky AlSulaiman, Badi A. Alotaibi, Abdulrahman Alshalani, Abdulaziz M. Almuqrin, Rehab B. Albakr, Jehad A. Aldali

**Affiliations:** 1 Chair of Medical and Molecular Genetics Research, Department of Clinical Laboratory Sciences, College of Applied Medical Sciences, King Saud University, Riyadh 12372, Saudi Arabia; halsudais@ksu.edu.sa (H.A.);; 2 Department of Clinical Laboratory Sciences, College of Applied Medical Sciences, King Saud University, Riyadh 12372, Saudi Arabia; 3 Department of Clinical Laboratory Sciences, College of Applied Medical Sciences, King Saud Bin Abdulaziz University for Health Sciences, Riyadh 11481, Saudi Arabia; 4 King Abdullah International Medical Research Center, Riyadh 11481, Saudi Arabia; 5 Department of Medicine, Division of Nephrology, College of Medicine, King Saud University, Riyadh 11472, Saudi Arabia; 6 Department of Pathology, College of Medicine, Imam Mohammad Ibn Saud Islamic University (IMSIU), Riyadh 13317, Saudi Arabia; jaaldali@imamu.edu.sa

**Keywords:** diabetes mellitus, type II, SGLT2 inhibitors, dapagliflozin, Saudi diabetic patients, renal profile, glycemic markers, CBC, lipid profile, liver enzymes

## Abstract

**Background**: This retrospective study evaluated the short-term effects of the SGLT2 inhibitor Dapagliflozin on laboratory markers in patients with type 2 diabetes mellitus (T2DM) over six months. **Methods**: Data were obtained from electronic health records at the King Abdullah International Medical Research Centre in Riyadh, Saudi Arabia. The study focused on adult T2DM patients who began Dapagliflozin treatment in 2023 or 2024 and underwent laboratory tests before, and six months after, starting treatment, resulting in 783 patients meeting the inclusion criteria. **Results**: Analysis revealed significant reductions in glycemic markers (*p*-value < 0.01). Hematological responses revealed divergent trends by sex: females showed increases in mean corpuscular hemoglobin concentration (MCHC) and red cell distribution width (RDW) (*p*-value < 0.01), while males showed a marginal decrease in mean corpuscular hemoglobin (MCH) (*p*-value < 0.05). Mean corpuscular volume (MCV) significantly decreased for both sexes (*p*-value < 0.01). Renal assessments indicated a significant increase in sodium levels and a marginal decrease in eGFR in males (*p*-values < 0.001 and <0.05, respectively), and elevated blood urea nitrogen (BUN) in both sexes (*p*-value < 0.01). Hepatic markers showed a marginal increase in alkaline phosphatase (ALP) in both sexes, with a trend toward a reduction in aspartate aminotransferase (AST) in males (*p*-value < 0.05). No significant changes were noted in lipid profiles or other hepatic markers. **Conclusions**: Dapagliflozin treatment in T2DM patients results in significant improvements in glycemic control and alterations in certain laboratory markers, indicating its potential impact on metabolic outcomes. Further research is needed to explore the underlying mechanisms and long-term effects.

## 1. Introduction

Type 2 diabetes mellitus (T2DM) is a chronic metabolic disorder characterized by hyperglycemia, resulting from insulin resistance and beta-cell dysfunction. It is among the fastest-growing health challenges worldwide, with significant morbidity and mortality attributed primarily to its vascular complications [1]. The latest International Diabetes Federation (IDF) Diabetes Atlas (2025) estimates that approximately 589 million adults are currently living with diabetes globally, the vast majority (over 90%) of whom have T2DM [2]. Alarmingly, projections indicate that this number will rise to 853 million by 2050 if current trends continue [2]. The increasing prevalence is attributed to urbanization, sedentary lifestyles, dietary changes, obesity epidemics, and an aging population [3].

Saudi Arabia is recognized as one of the countries with the highest rates of Type 2 Diabetes Mellitus (T2DM) globally, with estimates suggesting that approximately 25% of the adult population is impacted [4]. This elevated prevalence imposes considerable strains on both the healthcare system and society at large, as complications associated with diabetes, including cardiovascular disease, nephropathy, retinopathy, and neuropathy, lead to significant disability and increased mortality rates [5]. Achieving early and persistent glycemic control is essential for minimizing these complications, which is a conclusion supported by pivotal studies such as the Diabetes Control and Complications Trial (DCCT) and the UK Prospective Diabetes Study (UKPDS) [6,7].

The pharmacological treatment of T2DM has undergone significant developments in recent decades. In addition to conventional agents aimed at regulating insulin secretion or action, sodium-glucose cotransporter-2 (SGLT2) inhibitors have arisen as innovative therapeutic approaches with distinct mechanisms [8]. SGLT2 inhibitors, such as dapagliflozin, empagliflozin, and canagliflozin, selectively act on the proximal tubules of the kidney by inhibiting SGLT2 receptors, which account for roughly 90% of renal glucose reabsorption [9]. By disrupting this reabsorption, these medications enhance the excretion of glucose in urine, subsequently reducing plasma glucose concentrations through mechanisms that are independent of insulin pathways [10,11]. This mechanism presents benefits for individuals with impaired beta-cell function or insulin resistance.

In addition to their glucose-reducing properties, SGLT2 inhibitors exhibit a range of pleiotropic effects that have been shown to yield significant benefits for cardiovascular and renal health outcomes. Extensive clinical trials have demonstrated their ability to decrease hospitalizations for heart failure, the advancement of diabetic kidney disease, and the occurrence of significant adverse cardiovascular events [12,13]. Moreover, these drugs impact hematological parameters, such as elevations in hematocrit and erythropoietic indicators [14]. Biochemical markers such as liver enzymes, renal function tests, and inflammatory cytokines have demonstrated positive alterations with SGLT2 inhibitor treatment, further emphasizing their systemic advantages [15].

Despite growing global evidence, data on the effects of SGLT2 inhibitors specific to populations in Saudi Arabia remain limited. Differences in genetics, lifestyle, diet, and comorbidity prevalence may potentially influence drug responses and side effect profiles. Additionally, sex-based variations in response to SGLT2 inhibitors are poorly understood in this population. Understanding the short-term biochemical, hematological, and glycemic effects of these drugs is vital for optimizing patient care, including titration, monitoring, and managing adverse events during the critical initial treatment period.

The primary goal of this study was to evaluate the six-month effects of dapagliflozin on glycemic control, hematological parameters, renal function, and liver enzymes in Saudi males and females with T2DM. By discerning the early physiological responses and sex-specific differences, our findings aim to add valuable insights that can guide tailored clinical management and improve outcomes in this high-risk population.

## 2. Methods

### 2.1. Study Design

This retrospective study was conducted to evaluate the short-term effects of the SGLT2 inhibitor, Dapagliflozin, on hematological and biochemical markers in patients diagnosed with type 2 diabetes mellitus who initiated Dapagliflozin treatment in 2023 or 2024. The study was conducted using data collected from the electronic health records system at the King Abdullah International Medical Research Centre (KAIMRC), Ministry of National Guard, Health Affairs, Riyadh, Saudi Arabia, spanning the period from December 2022 to May 2025. The specific indication for initiating SGLT2 therapy—whether for kidney protection or for the treatment of T2DM—was not consistently documented in the available records.

The study included 4114 adult participants diagnosed with T2DM who had been prescribed Dapagliflozin for a minimum duration of one year, as verified through their medical records. Inclusion criteria included only patients with available hematological or biochemical laboratory tests before the start of treatment (up to −60 days) and who subsequently underwent a follow-up test six months later, allowing for a variability of ±60 days for this time point. The offset time median ranged from −3 to −13 days for the pre-treatment time point, and from −1 to −4 days for the post-treatment time point for all laboratory tests except the liver profile. The offset time for the liver profile test had a median ranging from −3 to −4 for the pre-treatment time point and +16 to +19 for the post-treatment time point. Exclusion criteria included patients with incomplete data, those who discontinued treatment prior to reaching the 6 month time point, and individuals under 18 years of age. Ultimately, the study sample consisted of 783 adult patients with a confirmed diagnosis of T2DM, each having been assessed for at least one biochemical marker or the hematological markers.

### 2.2. Patient Data

All laboratory tests were performed in a College of American Pathologists (CAP)-accredited laboratory. The hematological markers included the complete blood counts: red blood cell count (RBC), hematocrit (Hct), hemoglobin (Hgb), mean corpuscular hemoglobin (MCH), mean corpuscular hemoglobin concentration (MCHC), mean corpuscular volume (MCV), red cell distribution width (RDW), platelet count (PLT), and white blood cell count (WBC).

In terms of biochemical markers, the evaluation covered glycemic indicators such as hemoglobin A1C (HgbA1C), fasting blood glucose (FBG), and random blood glucose. The renal profile included creatinine, estimated glomerular filtration rate (eGFR), albumin, blood urea nitrogen (BUN), uric acid, sodium, potassium, and calcium levels. The liver profile comprised alanine transaminase (ALT), aspartate aminotransferase (AST), alkaline phosphatase (ALP), gamma-glutamyl transferase (GGT), and total bilirubin. Additionally, the lipid profile was analyzed through total cholesterol, triglycerides, high-density lipoprotein (HDL), and low-density lipoprotein (LDL) levels.

Demographic and clinical variables, such as sex, age, treatment history, and comorbidities, were extracted from the patients’ medical records. This study received ethical approval from the Institutional Review Board at KAIMRC (IRB-KAIMRC:IRB/2544/23 and study number: NRC23R/558/09), dated in October 2023.

### 2.3. Statistical Analysis

Data analysis was performed using SPSS software (version 26; IBM Corp., Armonk, NY, USA) for statistical testing and GraphPad Prism (version 10.4.1; GraphPad Software, San Diego, CA, USA) for data visualization. Descriptive statistics for all laboratory parameters, including both significant and non-significant findings, are presented in Supplementary Table S1 to complement the graphical summaries in the Results section. Categorical variables were summarized as frequencies (*n*) and percentages (%), while continuous variables were expressed as medians with interquartile ranges (IQR; Q1–Q3). Since differences between sex groups (male vs. female) are outside the scope of this study, the analysis primarily focused on changes within each sex group. For within-group comparisons stratified by sex, normally distributed data were analyzed using the paired Student’s *t*-test, whereas the Wilcoxon signed-rank test was applied for non-normally distributed data. To minimize the risk of Type I error associated with multiple comparisons across several laboratory parameters, we applied a more conservative significance level. A two-tailed *p*-value < 0.01 was considered statistically significant.

## 3. Results

### 3.1. Study Population

Table 1 provides a detailed overview of the characteristics and main comorbidities of 783 patients diagnosed with type 2 diabetes mellitus included in the study, composed of 384 females and 399 males, which indicates a balanced distribution between the two sexes. The median age for female patients was 64 years, while the male patients had a median age of 65 years. All participants were prescribed Dapagliflozin, with a significant proportion having had a history of metformin prescription—89.1% of females and 85.5% of males. Most patients were also on statin therapy (97.4% of females and 97.0% of males), with a considerable portion having a history of receiving steroid treatment, particularly among females (82.6% compared to 68.2% of males). In terms of comorbid conditions, both groups showed a high prevalence of hypertension, affecting 76.3% of females and 68.9% of males. Chronic ischemic heart disease was more prevalent among male participants (52.6% vs. 31.5%). Other notable comorbidities illustrated in Table 1 were chronic kidney disease (CKD), which is defined as abnormalities of kidney structure or function that are present for at least 3 months and have implications for health, as per the Kidney Disease Improving Global Outcomes (KDIGO) guidelines [16], and heart failure, affecting 16.1% and 31.5% of females and 20.8% and 28.1% of males, respectively. Acute myocardial infarction (MI) was reported in 12.0% of females and 20.1% of males. Stroke incidences were comparable between genders, affecting 12.0% of females and 13.5% of males. Rare conditions, such as systemic lupus erythematosus (SLE), were present in a minimal number of patients, with 0.5% of females and 0.3% of males affected. Chronic hepatitis was noted in only two male participants (0.5%). Overall, the patient profiles illustrate a diverse range of underlying comorbidities and treatment histories, characteristic of a population managing complex health conditions like T2DM. The comprehensive list of all recorded comorbidities is provided in the Supplementary Table S2 for further review.

### 3.2. Changes in Glycemic Markers Following SGLT2 Inhibitor Treatment in Diabetic Patients

**Figure 1:**
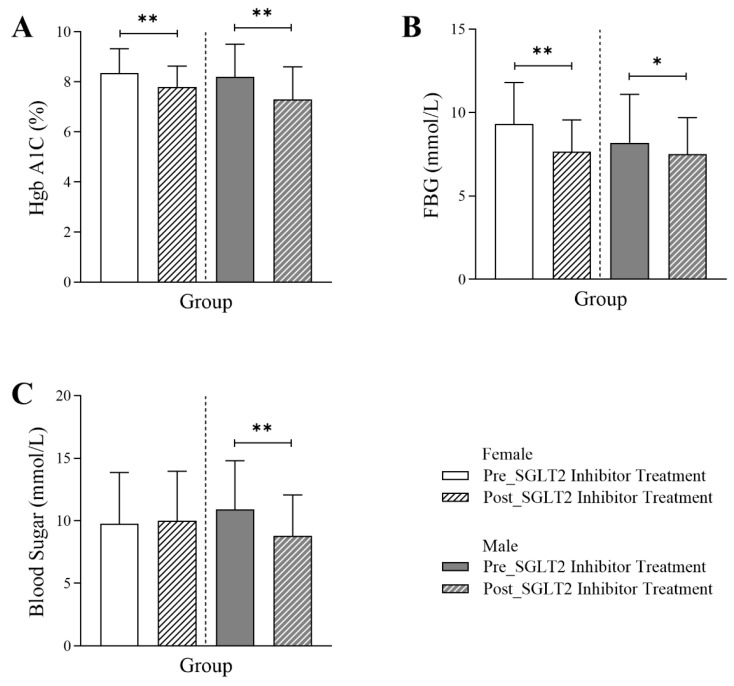
Differences in glycemic markers in female and male T2DM patients before and 6 months after the initiation of SGLT2 inhibitor treatment, including (**A**) HbA1C% (*n* = 255, 110 female and 115 male), (**B**) fasting blood glucose (FBG) (*n* = 191, 88 female and 103 male), and (**C**) random blood glucose (*n* = 452, 232 female and 220 male). The statistical significance levels are indicated (* *p* < 0.01, ** *p* < 0.001).

Figure 1 illustrates that, in both male and female patients diagnosed with type 2 diabetes, the glycemic markers HbA1c and fasting blood glucose (FBG) were significantly lower six months post-SGLT2 inhibitor treatment. The data indicate a marked reduction in HbA1c levels in female patients post-treatment (7.8 Q1–Q3: 7.1–8.63) compared to the same patients before the initiation of the treatment (8.35 Q1–Q3: 7.38–9.33; *p*-value < 0.001). Similarly, in male patients, HbA1c levels dropped significantly post-treatment (7.3 Q1–Q3: 6.5–8.6) compared to pre-treatment values (8.2 Q1–Q3: 7.1–9.5; *p*-value < 0.001). The FBG values in females and males were reduced significantly post-treatment (female: 7.65 Q1–Q3: 6.1–9.56; male: 7.5 Q1–Q3: 6.6–9.7) compared to the readings before treatment (female: 9.3 Q1–Q3: 7.1–11.8; *p*-value < 0.001, male: 8.2 Q1–Q3: 6.6–11.1; *p*-value < 0.01), collectively demonstrating a clear effect of SGLT2 inhibitors on glycemic control in this population. Additionally, a significant reduction in random blood glucose levels was observed exclusively in male patients (10.9 Q1–Q3: 7.3–14.8 vs. 8.8 Q1–Q3: 6.5–12.1; *p*-value < 0.001).

### 3.3. Changes in Hematological Markers Following SGLT2 Inhibitor Treatment in Diabetic Patients

**Figure 2:**
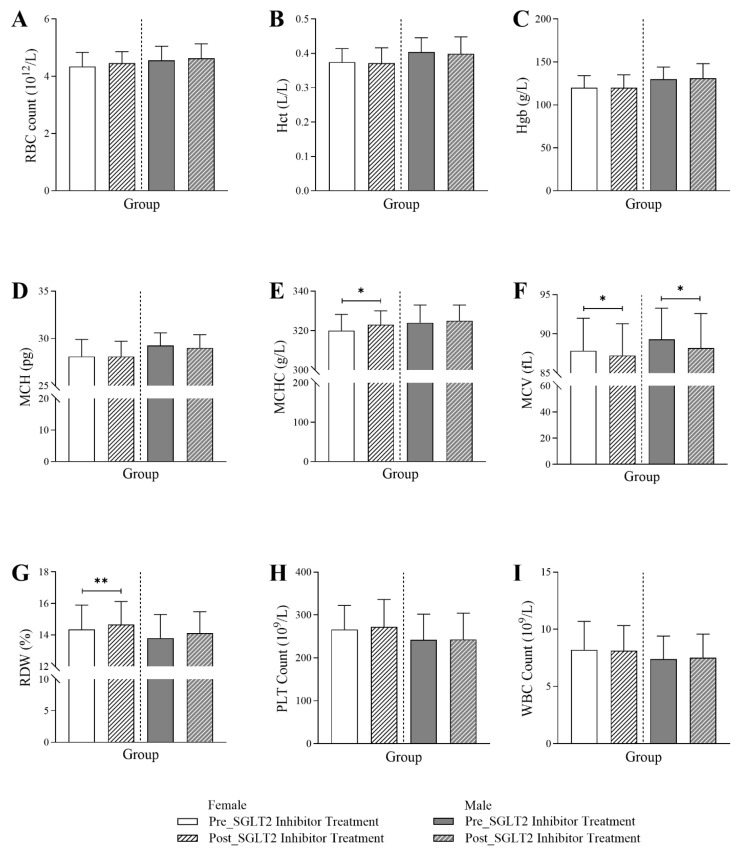
Changes in hematological markers in female and male T2DM patients before and 6 months after the initiation of SGLT2 inhibitor treatment (*n* = 594, 294 female and 300 male), including (**A**) red blood cell count (RBC), (**B**) hematocrit (Hct), (**C**) hemoglobin (Hgb), (**D**) mean corpuscular hemoglobin (MCH), (**E**) mean corpuscular hemoglobin concentration (MCHC), (**F**) mean corpuscular volume (MCV), (**G**) red cell distribution width (RDW), (**H**) platelet count (PLT), and (**I**) white blood cell count (WBC). The statistical significance levels are indicated (* *p* < 0.01, ** *p* < 0.001).

In Figure 2, a differential response to SGLT2 inhibitors was observed between male and female patients in their hematological profiles. While MCHC and RDW showed a significant increase post-treatment exclusively in female patients, MCH values trended toward a reduction only in male patients post-treatment. For both sexes, there was a significant reduction in MCV values post-treatment. The data showed that there was a significant increase following the initiation of the treatment in both MCHC (320 Q1–Q3: 314–328 vs. 323 Q1–Q3: 315–330; *p*-value < 0.01) and RDW (14.35 Q1–Q3: 13.2–15.9 vs. 14.65 Q1–Q3: 13.68–16.13; *p*-value < 0.001), only in female patients. For the MCH, there was a marginally significant reduction post-treatment (29.25 Q1–Q3: 27.4–30.6) compared to values before the treatment initiation (29 Q1–Q3: 27.1–30.4) in male patients (*p*-value < 0.05). The SGLT2 inhibitor treatment significantly reduced the MCV values in female patients (87.25 Q1–Q3: 81.58–91.3) and in male patients (88.2 Q1–Q3: 83.65–92.6) post-treatment compared to values before treatment (female: 87.85 Q1–Q3: 82.3–92; male: 89.3 Q1–Q3: 85.23–93.28) (*p*-value < 0.01). There were no significant changes in RBC, Hct, Hgb, PLT, or WBC for either gender.

### 3.4. Changes in Renal Profile Following SGLT2 Inhibitor Treatment in Diabetic Patients

**Figure 3:**
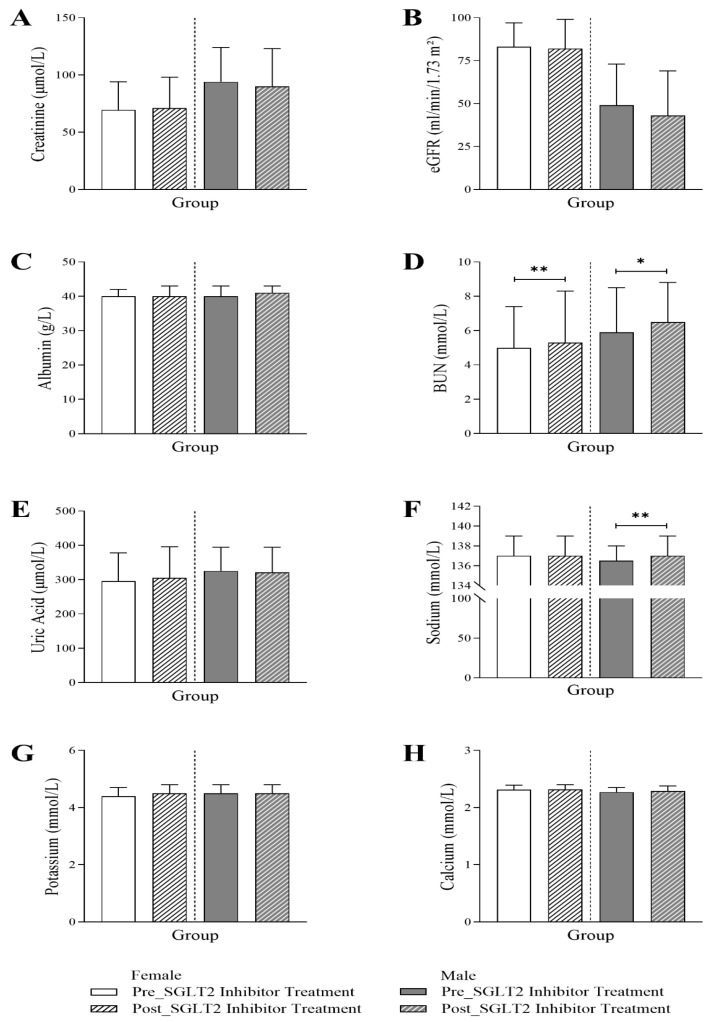
Changes in renal profile in female and male T2DM patients before and 6 months after the initiation of SGLT2 inhibitor treatment, including (**A**) creatinine (*n* = 735, 362 female and 373 male), (**B**) estimated glomerular filtration rate (eGFR) (*n* = 735, 362 female and 373 male), (**C**) albumin (*n* = 569, 289 female and 280 male), (**D**) blood urea nitrogen (BUN) (*n* = 696, 339 female and 357 male), (**E**) uric acid (*n* = 573, 295 female and 278 male), (**F**) sodium (*n* = 694, 338 female and 356 male), (**G**) potassium (*n* = 689, 333 female and 356 male), and (**H**) calcium levels (*n* = 570, 291 female and 279 male). The statistical significance levels are indicated (* *p* < 0.01, ** *p* < 0.001).

Figure 3 presents the assessment of the renal profile, showing an interesting trend towards a decrease in eGFR, only for male patients, following the initiation of SGLT2 inhibitor treatment (49 Q1–Q3: 28–73 vs. 43 Q1–Q3: 27.5–69; *p*-value < 0.05). At baseline, eGFR levels were lower in males compared to females (male vs. female 49 Q1–Q3: 28–73 vs. 83 Q1–Q3: 57.75–97). Post-treatment, both female and male patients exhibited a significant elevation in BUN (female: 5 Q1–Q3: 3.7–7.4 vs. 5.3 Q1–Q3: 4.1–8.3; *p*-value < 0.001, male: 5.9 Q1–Q3: 4.65–8.5 vs. 6.5 Q1–Q3: 5–8.8; *p*-value < 0.01). A similar trend was observed for sodium levels, with a more pronounced effect in males (female: 137 Q1–Q3: 134–139 vs. 137 Q1–Q3: 135–139; *p*-value < 0.05, male: 136 Q1–Q3: 134–138 vs. 137 Q1–Q3: 135–139; *p*-value < 0.001). There were no significant changes observed in creatinine, albumin, uric acid, potassium, or calcium.

### 3.5. Changes in Liver Profile Following SGLT2 Inhibitor Treatment in Diabetic Patients

**Figure 4:**
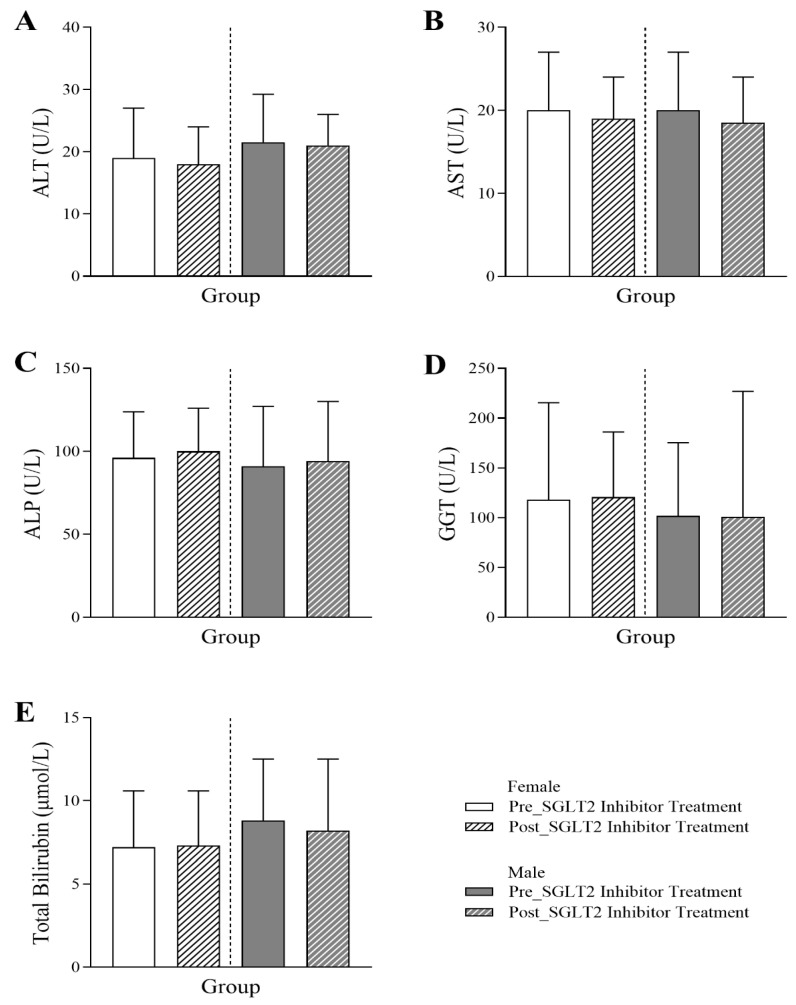
Changes in liver profile in female and male T2DM patients before and 6 months after the initiation of SGLT2 inhibitor treatment, including (**A**) alanine transaminase (ALT) (*n* = 225, 115 female and 110 male), (**B**) aspartate aminotransferase (AST) (*n* = 225, 115 female and 110 male), (**C**) alkaline phosphatase (ALP) (*n* = 587, 288 female and 299 male), (**D**) gamma-glutamyl transferase (GGT) (*n* = 77, 38 female and 39 male), and (**E**) total bilirubin (*n* = 589, 287 female and 297 male). The statistical significance levels are indicated.

Figure 4 illustrates the evaluation of the effect of SGLT2 inhibitor treatment on the liver profile. Following the SGLT2 inhibitor treatment, there was an increasing trend in ALP levels for both female and male patients (female: 96 Q1–Q3: 74.25–123.75 vs. 100 Q1–Q3: 78–126; male: 91 Q1–Q3: 69–127 vs. 94 Q1–Q3: 70–130) (*p*-value < 0.05). A marginal decrease in AST levels was only observed in males (20 Q1–Q3: 17–27 vs. 18.5 Q1–Q3: 15–24) (*p*-value < 0.05). No significant effects were noted in ALT, GGT, or total bilirubin levels post-treatment.

### 3.6. Assessment of SGLT2 Inhibitors on Lipid Indices in Diabetic Patients

**Figure 5:**
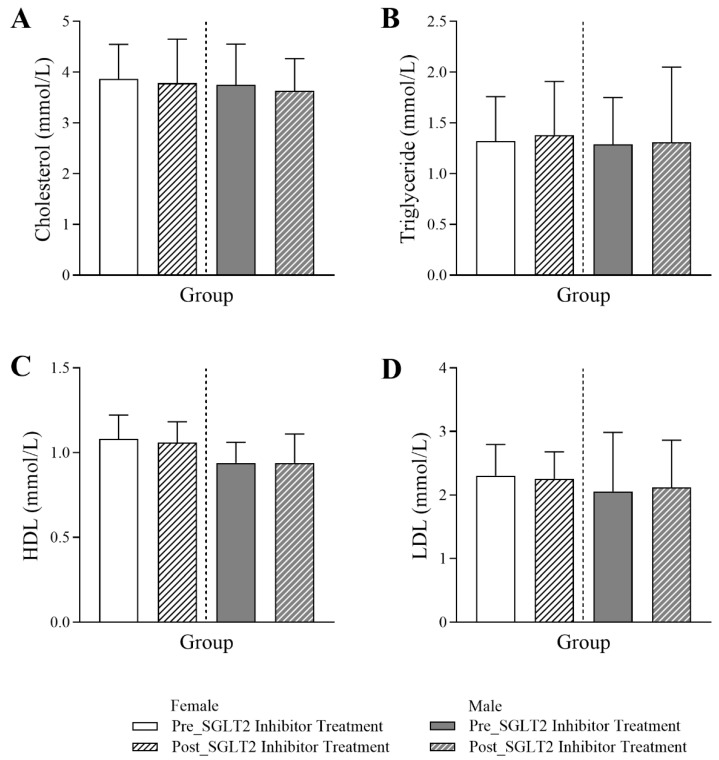
Assessment of SGLT2 inhibitors on lipid indices in female and male T2DM patients before and 6 months after the initiation of SGLT2 inhibitor treatment, including (**A**) cholesterol (*n* = 275, 126 female and 149 male), (**B**) triglycerides (*n* = 230, 103 female and 127 male), (**C**) high-density lipoprotein (HDL) (*n* = 275, 126 female and 149 male), and (**D**) low-density lipoprotein (LDL) levels (*n* = 274, 126 female and 148 male). The statistical significance levels are indicated.

Figure 5 indicates that SGLT2 inhibitor treatment did not lead to any significant changes in the lipid profile for both male and female diabetic patients.

## 4. Discussion

The worldwide prevalence of type 2 diabetes mellitus (T2DM) is steadily rising, presenting considerable clinical challenges not only due to high blood glucose levels but also because of the complex interaction of various comorbid conditions that worsen patient outcomes. As new therapies are developed, it is crucial to understand their comprehensive physiological effects as measured by laboratory tests, beyond merely lowering glucose to tailor treatment effectively. This study sought to investigate the diverse effects of dapagliflozin, an SGLT2 inhibitor, on blood glucose control, complete blood count, kidney function, liver markers, and lipid profile in Saudi male and female T2DM patients, uncovering divergent trends in the effects of treatment between sexes in this population.

Our findings strongly support the well-established effectiveness of SGLT2 inhibitors in lowering glycemic indicators. Both female and male patients showed statistically significant decreases in HbA1c and fasting blood glucose after six months of treatment. These results are consistent with numerous large randomized controlled trials and meta-analyses reporting comparable reductions in blood sugar levels, underscoring the powerful glucose-lowering effects of dapagliflozin and similar drugs [17,18,19].

A more thorough analysis of the sex-specific hematological alterations observed after SGLT2 inhibitor treatment indicates several reliable mechanisms. In our research, female patients showed increases in MCHC and RDW, whereas male patients demonstrated a trending decrease in MCH. These apparent differences may indicate fundamental biological variations in erythrocyte hydration levels and red cell turnover between sexes. For example, SGLT2 inhibitors have been associated with heightened erythropoietin production and enhanced renal oxygenation—factors that can promote erythropoiesis and alter red cell indices [20]. Nevertheless, the current literature suggests that there may be differences in kidney responses and erythrocyte dynamics between women and men during SGLT2i therapy, with some studies indicating that women experience reduced cardiovascular and metabolic advantages compared to men, possibly due to hormonal influences on renal and hematological functions [21]. Studies indicate that increases in MCHC may result from slight variations in cellular hydration or modifications in membrane permeability, potentially signifying a sex-specific adaptation to osmotic stress due to SGLT2 inhibition [22]. In contrast, the observed trending decline in MCH among males could suggest heightened red cell turnover or changes in hemoglobin synthesis that differ from the female response. Additionally, homocysteine levels and cardiovascular risk factors have been linked to MCV and MCH, implying that metabolic profiles affect red cell indices in a manner that is dependent on sex [23].

The clinical significance of these findings has yet to be completely clarified. Nevertheless, the recognition of sex-divergent trends highlights the necessity for more detailed methods in tracking hematological parameters and tailoring diabetes treatment. Subsequent research should incorporate molecular analyses to elucidate the effects of SGLT2 inhibitors on erythrocyte physiology and patient outcomes across different genders [24].

In terms of the effect of SGLT2 inhibitors on kidney functions, our findings showed a significant increase in sodium levels and a marginal decrease in eGFR after treatment in male patients, while both sexes exhibited increases in BUN levels. These observations align with the initial hemodynamic effects of SGLT2 inhibitors, which involve constriction of the afferent arteriole and a drop in intraglomerular pressure—these are mechanisms believed to cause the temporary eGFR decline seen in clinical settings [19,20]. Importantly, these early changes are thought to precede the long-term kidney protection demonstrated in large outcome trials. Our results also support retrospective studies indicating that renal responses vary by sex and baseline kidney function, highlighting the need for individualized kidney monitoring when starting SGLT2 inhibitor treatment [25,26,27]. Moreover, it is essential to consider the baseline eGFR values, as the observed differences, with males having a lower median eGFR compared to females, can contribute to the observed sex-specific eGFR responses. This substantial baseline difference necessitates careful interpretation of the findings, particularly regarding whether the observed eGFR decline trend is a reversible hemodynamic phenomenon or indicative of an underlying sex-specific divergence in renal trajectory.

A notable trending rise in ALP levels was seen in both genders following the SGLT2 inhibitor treatment; however, these levels stayed within the normal physiological range. This indicates that the observed changes are likely not pathological but may represent slight modifications in hepatic or bone metabolism instead of clear liver damage. It is crucial to understand that while ALP is frequently part of liver function tests, it is not specific to liver steatosis or inflammation and can be affected by various extrahepatic factors, including bone metabolism [28]. Consequently, the clinical significance of these alterations remains unclear without corroborating imaging or histological evidence for non-alcoholic fatty liver disease (NAFLD). Our results are consistent with meta-analyses that indicate a generally neutral or marginally positive impact of SGLT2 inhibitors on liver parameters, which supports their favorable safety profile. Nonetheless, additional research employing direct assessments of hepatic steatosis is necessary [29]. The lack of statistically significant changes in liver profile levels supports previous safety data, confirming that these drugs have a neutral-to-positive impact on liver health. These findings align with growing evidence indicating that SGLT2 inhibitors may have liver-protective effects, potentially by reducing hepatic steatosis, inflammation, or fibrosis in patients with T2DM and NAFLD [15]. Differences observed in some studies might be due to variations in study populations, the drug used, treatment durations, or the presence of other health conditions [30].

Our cohort’s high prevalence of common T2DM-associated comorbidities, including hypertension, chronic ischemic heart disease, chronic kidney disease, and heart failure, mirrors findings in comparable epidemiological datasets [31,32,33,34,35]. The observed sex differences, particularly the higher rates of ischemic heart disease and myocardial infarction in males, resonate with well-established gender discrepancies in cardiovascular risk profiles among diabetic populations [36,37,38]. These comorbidities may contribute to the observed changes and emphasize the clinical complexity often encountered, highlighting the need for comprehensive, multifactorial treatment approaches. Further investigation and long-term studies are essential to provide a clearer understanding of the effects of SGLT2 treatment in the context of these comorbidities.

With all the strengths of this study, it is also important to acknowledge the limitations that warrant attention. The observational, single-center design may introduce selection bias and limit the generalizability of findings to broader populations. Notably, while our study initially investigated 4114 patients, only approximately 20% met the inclusion criteria, which may influence the representativeness of our sample. This reduction in patient numbers underscores the need for caution in applying our findings to different populations and clinical settings. The six-month follow-up window, while adequate to detect short-term changes, may not fully capture the long-term renal, hepatic, and hematological trajectories influenced by SGLT2 inhibitors. Confounding factors—such as variable concomitant medications, lifestyle factors, and unmeasured comorbidities—could also affect outcomes. The substantial proportion of patients with comorbidities like chronic kidney disease and heart failure aligns with the existing literature and underscores the real-world applicability of our study outcomes, at least in the Saudi population [31,32,33,34,35]. However, we acknowledge that these comorbidities may affect the generalizability of our findings, warranting careful consideration when applying our results to broader populations. Furthermore, the existence of potential concomitant medications like statins and steroids adds another level of complexity, given that these drugs can also affect outcomes, thereby complicating the ability to attribute laboratory changes exclusively to dapagliflozin. Given the retrospective nature of our study and the limited availability of comprehensive data, we acknowledge that we cannot fully address these potential confounders in our analysis. Additionally, although we highlighted divergent trends by sex in our findings, our statistical analysis plan focused primarily on within-group changes and did not formally test sex differences. This methodological choice limits our ability to conclusively state that responses differ significantly between males and females, and further studies are needed. Future research should involve larger, multi-center cohorts with randomized designs and longer observation periods to validate and extend our insights, particularly regarding sex-specific differences and mechanistic underpinnings.

This research underscores the effectiveness of dapagliflozin in attaining significant glycemic control in patients with T2DM, while revealing new sex-dependent influences on hematological and renal parameters. These results enhance the comprehension of the pleiotropic effects of SGLT2 inhibitors and emphasize the necessity for personalized treatment approaches. Additional longitudinal and mechanistic investigations are needed to refine therapeutic strategies and enhance patient outcomes in this widely prevalent condition.

## 5. Conclusions

The use of Dapagliflozin in patients with T2DM results in significant enhancements in glycemic control and notable impacts on various laboratory markers. This suggests that Dapagliflozin offers beneficial effects on metabolic health, reinforcing its role in managing T2DM. Additionally, the treatment appears to have different effects on hematological and biochemical markers, with variations that may be influenced by sex. These outcomes highlight the importance of considering sex differences when evaluating the treatment’s effects on metabolic and laboratory parameters in T2DM. To build on these findings, future prospective studies should investigate the long-term impacts and include comprehensive testing to elucidate the underlying mechanisms of these responses.

## Figures and Tables

**Table 1:** Characteristics and main comorbidities of the patients included in the study.

Variable	Group	
	Female (*n* = 384)	Male (*n* = 399)
**Age** (years), median (IQR)	64 (56–71)	65 (56–73)
**History of Treatment**, *n* (%)		
Dapagliflozin	384 (100)	399 (100)
Metformin	342 (89.1)	341 (85.5)
Statin	374 (97.4)	387 (97.0)
Steroids	317 (82.6)	272 (68.2)
**Comorbidity**, *n* (%)		
T2DM	384 (100)	399 (100)
CKD	62 (16.1)	83 (20.8)
Acute MI	46 (12.0)	80 (20.1)
Chronic Ischaemic HD	121 (31.5)	210 (52.6)
Chronic Hepatitis	0 (0.0)	2 (0.5)
Heart Failure	121 (31.5)	112 (28.1)
Hypertension	293 (76.3)	275 (68.9)
Stroke	46 (12.0)	54 (13.5)
SLE	2 (0.5)	1 (0.3)

*n*; frequencies, %; percentages, IQR; interquartile range, T2DM: type 2 diabetes mellitus, CKD; chronic kidney disease, MI; myocardial infarction, HD; heart disease, and SLE; systemic lupus erythematosus.

## Data Availability

The data presented in this study can be obtained upon reasonable request from the corresponding author, subject to the hospital’s policy approval.

## Supplementary Materials

The following supporting information can be downloaded at: https://www.mdpi.com/article/10.3390/jcm14227985/s1, Table S1: Descriptive analyses for laboratory parameters and Table S2: Comorbidity of patients included in the study.
