# Supplementary material for: Short-Term Laboratory Outcomes of SGLT2 Inhibitor Use in Type 2 Diabetic Patients: A Retrospective Analysis
Source: J Clin Med. 2025 Nov 11;14(22):7985. doi: 10.3390/jcm14227985 (PMC12653581; doi:10.3390/jcm14227985)
Supplement: Supplementary file 1 [file jcm-14-07985-s001.zip › jcm-3957637-supplementary.pdf]

Supplementary Table S1. Descriptive analyses for Laboratory parameters

Parameter	Groups			
	Female		Male	
	Pre-SGLT2	Post-SGLT2	Pre-SGLT2	Post-SGLT2
**Hgb A1C (%)**				
Number of values	110	110	115	115
25% Percentile	7.38	7.10	7.10	6.50
Median	8.35	7.80	8.20	7.30
75% Percentile	9.33	8.63	9.50	8.60
Mean	8.55	7.93	8.37	7.60
Standard Deviation	1.75	1.36	1.76	1.41
**FBG (mmol/L)**				
Number of values	88	88	103	103
25% Percentile	7.10	6.10	6.60	6.60
Median	9.30	7.65	8.20	7.50
75% Percentile	11.80	9.56	11.10	9.70
Mean	10.11	8.62	9.15	8.19
Standard Deviation	4.82	4.12	3.79	2.88
**Blood Sugar (mmol/L)**				
Number of values	232	232	220	220
25% Percentile	7.23	7.70	7.30	6.50
Median	9.75	10.00	10.90	8.80
75% Percentile	13.85	13.95	14.80	12.08
Mean	11.18	11.21	12.21	9.99
Standard Deviation	5.65	5.50	6.56	4.71
**RBC (10^{12}/L)**				
Number of values	294	294	300	300
25% Percentile	3.89	3.82	3.97	3.92
Median	4.34	4.46	4.56	4.63
75% Percentile	4.84	4.86	5.05	5.14
Mean	4.31	4.33	4.48	4.52
Standard Deviation	0.73	0.74	0.81	0.86
**Hct (%)**				
Number of values	294	294	300	300
25% Percentile	0.33	0.33	0.34	0.34
Median	0.38	0.37	0.40	0.40
75% Percentile	0.41	0.42	0.45	0.45
Mean	0.37	0.37	0.40	0.39
Standard Deviation	0.06	0.06	0.07	0.08
**Hgb (g/L)**				
Number of values	294	294	300	300
25% Percentile	107.75	106.00	111.25	110.25
Median	120.00	120.00	130.00	131.00
75% Percentile	134.00	135.00	144.00	148.00
Mean	119.60	119.95	128.20	128.50
Standard Deviation	19.33	19.90	23.54	25.80

**MCH (pg)**				
Number of values	294	294	300	300
25% Percentile	26.18	26.10	27.40	27.10
Median	28.10	28.10	29.25	29.00
75% Percentile	29.90	29.70	30.60	30.40
Mean	27.99	27.87	28.77	28.51
Standard Deviation	2.94	2.87	2.71	2.74
**MCHC (g/L)**				
Number of values	294	294	300	300
25% Percentile	314.00	315.00	316.00	318.00
Median	320.00	323.00	324.00	325.00
75% Percentile	328.25	330.00	333.00	333.00
Mean	321.43	323.38	325.08	325.97
Standard Deviation	10.63	11.36	11.67	11.34
**MCV (fL)**				
Number of values	294	294	300	300
25% Percentile	82.30	81.58	85.23	83.65
Median	87.85	87.25	89.30	88.20
75% Percentile	92.00	91.30	93.28	92.60
Mean	87.08	86.17	88.49	87.41
Standard Deviation	8.17	8.03	7.88	7.65
**RDW (%)**				
Number of values	294	294	300	300
25% Percentile	13.20	13.68	13.10	13.20
Median	14.35	14.65	13.80	14.10
75% Percentile	15.90	16.13	15.30	15.48
Mean	14.73	15.24	14.49	14.69
Standard Deviation	2.14	2.37	2.33	2.37
**PLT Count (10^9/L)**				
Number of values	294	294	300	300
25% Percentile	217.00	213.75	195.00	192.00
Median	266.00	272.00	242.00	243.00
75% Percentile	322.00	336.00	301.75	304.00
Mean	277.22	282.12	254.07	254.17
Standard Deviation	92.06	96.80	86.69	90.46
**WBC (10^9/L)**				
Number of values	294	294	300	300
25% Percentile	6.38	6.11	6.03	6.05
Median	8.19	8.13	7.40	7.51
75% Percentile	10.70	10.33	9.41	9.58
Mean	9.09	9.17	8.26	8.15
Standard Deviation	4.20	8.32	3.82	3.17
**Creatinine (µmol/L)**				
Number of values	362	362	373	373
25% Percentile	62.00	59.00	77.00	74.50
Median	69.50	71.00	94.00	90.00
75% Percentile	94.00	89.00	124.00	123.00

Mean	85.06	86.16	113.61	113.77
Standard Deviation	49.27	44.27	76.98	77.14
**eGFR (mL/min/1.73m^2)**				
Number of values	362	362	373	373
25% Percentile	57.75	54.00	28.00	27.50
Median	83.00	82.00	49.00	43.00
75% Percentile	97.00	99.00	73.00	69.00
Mean	77.09	75.99	51.94	49.18
Standard Deviation	25.56	26.86	24.38	24.26
**Albumin (g/L)**				
Number of values	289	289	280	280
25% Percentile	37.00	37.00	37.00	38.00
Median	40.00	40.00	40.00	41.00
75% Percentile	42.00	43.00	43.00	43.00
Mean	39.51	39.37	39.55	39.82
Standard Deviation	4.57	4.77	5.05	5.17
**BUN (mmol/L)**				
Number of values	339	339	357	357
25% Percentile	3.70	4.10	4.65	5.00
Median	5.00	5.30	5.90	6.50
75% Percentile	7.40	8.30	8.50	8.80
Mean	6.17	7.21	7.20	7.83
Standard Deviation	3.69	5.02	4.06	4.71
**Uric Acid (µmol/L)**				
Number of values	295	295	278	278
25% Percentile	233.00	323.00	257.00	266.00
Median	296.00	305.00	325.00	321.50
75% Percentile	378.00	396.00	394.25	394.50
Mean	314.51	325.30	335.36	336.88
Standard Deviation	107.32	118.45	105.91	104.25
**Sodium (mmol/L)**				
Number of values	338	338	356	356
25% Percentile	134.00	135.00	134.00	135.00
Median	137.00	137.00	136.50	137.00
75% Percentile	139.00	139.00	138.00	139.00
Mean	136.44	136.96	136.15	136.91
Standard Deviation	4.11	3.67	3.48	3.38
**Potassium (mmol/L)**				
Number of values	333	333	356	356
25% Percentile	4.20	4.15	4.20	4.20
Median	4.40	4.50	4.50	4.50
75% Percentile	4.70	4.80	4.80	4.80
Mean	4.48	4.45	4.53	4.54
Standard Deviation	0.52	0.53	0.50	0.50
**Calcium (mmol/L)**				
Number of values	291	291	279	279
25% Percentile	2.23	2.23	2.19	2.19

Median	2.31	2.32	2.27	2.29
75% Percentile	2.39	2.40	2.35	2.38
Mean	2.31	2.30	2.26	2.28
Standard Deviation	0.14	0.13	0.14	0.15
**ALT (U/L)**				
Number of values	115	115	110	110
25% Percentile	14.00	13.00	17.00	16.00
Median	19.00	18.00	21.50	21.00
75% Percentile	27.00	24.00	29.25	26.00
Mean	25.38	22.81	25.65	23.54
Standard Deviation	23.77	19.32	16.75	14.37
**AST (U/L)**				
Number of values	115	115	110	110
25% Percentile	16.00	16.00	17.00	15.00
Median	20.00	19.00	20.00	18.50
75% Percentile	27.00	24.00	27.00	24.00
Mean	24.75	21.95	24.56	22.11
Standard Deviation	22.38	11.38	15.73	19.55
**ALP (U/L)**				
Number of values	288	288	299	299
25% Percentile	74.25	78.00	69.00	70.00
Median	96.00	100.00	91.00	94.00
75% Percentile	123.75	126.00	127.00	130.00
Mean	114.55	117.92	108.81	116.73
Standard Deviation	78.96	78.90	76.63	87.62
**GGT (U/L)**				
Number of values	38	38	39	39
25% Percentile	66.20	50.80	65.00	62.00
Median	118.00	120.75	102.00	100.90
75% Percentile	215.50	186.08	175.30	227.00
Mean	154.06	155.03	168.75	145.46
Standard Deviation	131.08	139.89	226.97	111.47
**Total Bilirubin (µmol/L)**				
Number of values	287	287	297	297
25% Percentile	5.30	5.10	6.30	6.20
Median	7.20	7.30	8.80	8.20
75% Percentile	10.60	10.60	12.50	12.50
Mean	9.48	9.58	10.88	12.24
Standard Deviation	7.48	10.04	7.49	25.16
**Cholesterol (mmol/L)**				
Number of values	126	126	149	149
25% Percentile	3.32	3.17	3.12	3.08
Median	3.87	3.79	3.75	3.63
75% Percentile	4.55	4.65	4.55	4.27
Mean	4.14	3.98	3.89	3.81
Standard Deviation	1.23	1.29	1.08	1.10
**Triglyceride (mmol/L)**				

Number of values	103	103	127	127
25% Percentile	1.06	0.95	0.97	1.00
Median	1.32	1.38	1.29	1.31
75% Percentile	1.76	1.91	1.75	2.05
Mean	1.49	1.59	1.53	1.63
Standard Deviation	0.75	1.07	0.93	1.36
**HDL (mmol/L)**				
Number of values	126	126	149	149
25% Percentile	0.93	0.88	0.80	0.81
Median	1.08	1.06	0.94	0.94
75% Percentile	1.22	1.18	1.06	1.11
Mean	1.09	1.07	0.95	0.97
Standard Deviation	0.29	0.28	0.23	0.22
**LDL (mmol/L)**				
Number of values	126	126	148	148
25% Percentile	1.87	1.69	1.67	1.64
Median	2.31	2.26	2.06	2.12
75% Percentile	2.80	2.68	2.99	2.86
Mean	2.50	2.33	2.38	2.32
Standard Deviation	1.04	1.01	1.04	0.96

FBG; Fasting blood glucose, RBC; red blood cell count, Hct; hematocrit, Hgb; hemoglobin, MCH; mean corpuscular hemoglobin, MCHC; mean corpuscular hemoglobin concentration, MCV; mean corpuscular volume, RDW; red cell distribution width, PLT; platelet count, WBC; white blood cell count, eGFR; estimated glomerular filtration rate, BUN; blood urea nitrogen, ALT; alanine transaminase, AST; aspartate aminotransferase, ALP; alkaline phosphatase, GGT; gamma-glutamyl transferase, HDL; high-density lipoprotein, and LDL; low-density lipoprotein.

Supplementary Table S2. Comorbidity of patients included in the study

Comorbidity	Groups	
	Female (n=384)	Male (n=399)
Acute MI, n (%)	46 (12.0)	80 (20.1)
Arthritis, n (%)	11 (2.9)	10 (2.5)
Asthma, n (%)	118 (30.7)	59 (14.8)
CKD, n (%)	62 (16.1)	83 (20.8)
COPD, n (%)	6 (1.6)	7 (1.8)
Celiac Disease, n (%)	0 (0.0)	0 (0.0)
Chronic Hepatitis, n (%)	0 (0.0)	2 (0.5)
Chronic Ischaemic HD, n (%)	121 (31.5)	210 (52.6)
CHD, n (%)	0 (0.0)	2 (0.5)
Crohn Disease, n (%)	0 (0.0)	1 (0.3)
Diabetes, n (%)	384 (100)	399 (100)
Epilepsy, n (%)	5 (1.3)	7 (1.8)
Fibrosis Cirrhosis, n (%)	15 (3.9)	18 (4.5)
Heart Failure, n (%)	121 (31.5)	112 (28.1)
Hypertension, n (%)	293 (76.3)	275 (68.9)
Multiple Sclerosis, n (%)	1 (0.3)	0 (0.0)
Osteoporosis, n (%)	27 (7.0)	2 (0.5)
Rheumatoid Arthritis, n (%)	7 (1.8)	5 (1.3)
SLE, n (%)	2 (0.5)	1 (0.3)
Stroke, n (%)	46 (12.0)	54 (13.5)
Ulcerative colitis, n (%)	2 (0.5)	2 (0.5)
Vitiligo, n (%)	0 (0.0)	1 (0.3)
Cancer, n (%)	57 (14.8)	56 (14.0)

n; frequencies, %; percentages, MI; myocardial infarction, CKD; chronic kidney disease, COPD: chronic obstructive pulmonary disease, HD; heart disease, CHD; congenital heart defect, SLE; systemic lupus erythematosus.
